# Donor bone-marrow CXCR4+ Foxp3+ T-regulatory cells are essential for costimulation blockade-induced long-term survival of murine limb transplants

**DOI:** 10.1038/s41598-020-66139-x

**Published:** 2020-06-09

**Authors:** Liqing Wang, Zhonglin Wang, Rongxiang Han, Arabinda Samanta, Guanghui Ge, L. Scott Levin, Matthew H. Levine, Wayne W. Hancock

**Affiliations:** 1 Division of Transplant Immunology, Department of Pathology and Laboratory Medicine, and Biesecker Center for Pediatric Liver Disease, Children’s Hospital of Philadelphia and University of Pennsylvania, Philadelphia, PA 19104 USA; 2 Department of Surgery, University of Pennsylvania, Philadelphia, Pennsylvania USA; 3 Department of Orthopaedic Surgery, Hospital of the University of Pennsylvania, Philadelphia, Pennsylvania USA; 4 Department of Surgery, Division of Plastic Surgery, Hospital of the University of Pennsylvania, Philadelphia, Pennsylvania USA; 5 Department of Surgery, Children’s Hospital of Philadelphia, Philadelphia, Pennsylvania USA

**Keywords:** Immunology, Transplant immunology, Allotransplantation

## Abstract

Vascularized composite allotransplantation (VCA) allows tissue replacement after devastating loss but is currently limited in application and may be more widely performed if maintenance immunosuppression was not essential for graft acceptance. We tested whether peri-transplant costimulation blockade could prolong VCA survival and required donor bone-marrow cells, given that bone-marrow might promote graft immunogenicity or graft-versus-host disease. Peritransplant CD154 mAb/rapamycin (RPM) induced long-term orthotopic hindlimb VCA survival (BALB/c->C57BL/6), as did CTLA4Ig/RPM. Surprisingly, success of either protocol required a bone-marrow-associated, radiation-sensitive cell population, since long-bone removal or pre-transplant donor irradiation prevented long-term engraftment. Rejection also occurred if Rag1−/− donors were used, or if donors were treated with a CXCR4 inhibitor to mobilize donor BM cells pre-transplant. Donor bone-marrow contained a large population of Foxp3+ T-regulatory (Treg) cells, and donor Foxp3+ Treg depletion, by diphtheria toxin administration to DEREG donor mice whose Foxp3+ Treg cells expressed diphtheria toxin receptor, restored rejection with either protocol. Rejection also occurred if CXCR4 was deleted from donor Tregs pre-transplant. Hence, long-term VCA survival is possible across a full MHC disparity using peritransplant costimulation blockade-based approaches, but unexpectedly, the efficacy of costimulation blockade requires the presence of a radiation-sensitive, CXCR4+ Foxp3+ Treg population resident within donor BM.

## Introduction

The widespread application of vascularized composite allotransplantation (VCA) is currently limited by various factors, including the perceived risks of maintenance immunosuppression with calcineurin inhibitors that involve increased risks of the development of infection, neurotoxicity, nephrotoxicity and carcinoma^1–4^. In addition, clinical studies show that organ transplant recipients receiving calcineurin inhibitor therapy have impairment of Foxp3 + T-regulatory (Treg) cells, whereas Treg function is preserved under rapamycin (RPM) therapy^5^. Experimentally, peri-transplant costimulation blockade (COB) alone, using CD154 monoclonal antibody (mAb) or CTLA4-Ig, or COB plus a brief course of RPM, can induce long-term, Treg-dependent survival of organ transplants^6–9^. Critically, allograft recipients of COB-based protocols can develop donor-specific tolerance and remain free from the long-term development of chronic allograft rejection without maintenance immunosuppression^6–9^. Hence, understanding whether peri-transplant therapies can also benefit VCA recipients and avoid maintenance calcineurin inhibitor-based immunosuppression is an important consideration in efforts to promote the broader application of VCA procedures and to improve the subsequent quality of life in VCA recipients.

We hypothesized that a clinically relevant protocol such as peri-transplant delivery of some form of COB plus RPM might promote long-term VCA survival by inhibiting the allo-activation of conventional T cells and allowing the development of Treg-dependent mechanisms that maintain allograft survival. Corollaries of this over-arching hypothesis are that potentially both donor and recipient Tregs might contribute to graft survival; that Tregs must prevent potent host responses to allogeneic skin that is typically considered highly immunogenic; and that therapeutic agents that negatively or positively affect Treg function might impair or further promote outcomes, respectively. We sought to test these concepts in murine limb transplant models that are ideally suited to mechanistic investigations.

## Results

### Peri-transplant CD154/DST/RPM induces long-term orthotopic VCA survival

**Figure 1:**
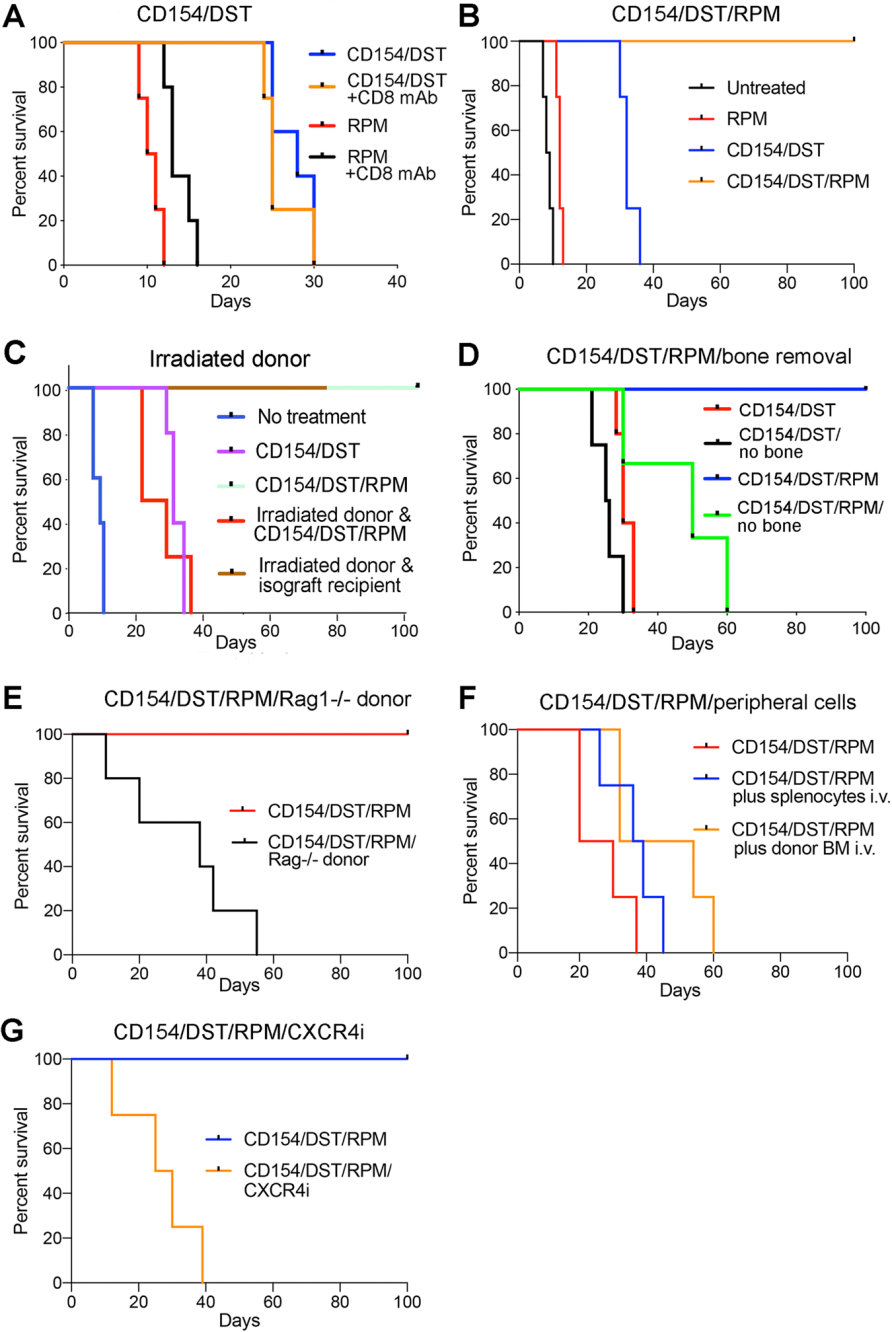
Peri-transplant CD154/RPM therapy induces long-term orthotopic hindlimb VCA survival; each panel involves 4–5 allografts/group and all studies were repeated at least once with comparable results. (**A**) Survival of allografts in mice treated with CD8 mAb therapy (once weekly until rejection) plus RPM was increased compared to use of RPM alone (*p < 0.05). Still better survival was achieved using CD154/DST at the time of engraftment (**p < 0.01 vs. RPM or RPM/CD8 mAb), but CD8 mAb did not significantly increase survival when combined with CD154/DST. (**B**) Addition of 30 days of RPM therapy from the time of engraftment to CD154/DST led to VCA survival of >100 d (**P < 0.01 vs. CD154/DST). (**C**) Donor irradiation led to subsequent impairment of orthotopic VCA survival despite optimal peritransplant therapy with CD154/DST/RPM; **p < 0.01 for irradiated allograft donor group vs. non-irradiated donor group; no significant difference between CD154/DST and CD154/DST/donor irradiation, whereas both latter groups showed prolongation (*p < 0.05) compared to untreated controls. (**D**) In contrast to allografts that survived>100 d in recipients treated with CD154/DST/RPM, grafts in which the bone was removed before transplantation into recipients treated with CD154/DST/RPM, underwent rejection (**p < 0.01). In the absence of any added RPM, allografts were rejected even faster (**p < 0.01) vs. CD154/DST/RPM (no bone). (**E**) Allografts from Rag1−/− donors were acutely rejected despite CD154/DST/RPM therapy (**p < 0.01). (**F**) Survival of allografts from Rag1−/− donors was improved by i.v. injection of donor splenocytes (p < 0.05) at the time of engraftment, and i.v. injection of donor bone-marrow cells at the time of engraftment had further benefit (*p < 0.05), but rejection could not be prevented despite CD154/DST/RPM therapy. (**G**) Use of CXCR4i therapy in donor mice on days −4, −2 and on the day of transplantation prevented long-term survival of subsequent hindlimb allografts despite therapy with CD154/DST/RPM (**p < 0.01).

Therapy with CD154 mAb (MR-1) plus donor splenocyte transfusion (DST, 5 × 10^6^ cells, i.v.) is very effective at inducing tolerance of various kinds of allografts, including that of hearts, kidneys or islets transplanted across fully MHC-disparate combinations such as BALB/c->C57BL/6 mice (H-2^d^->H-2^b^)^6,7^. However, this protocol, while able to prolong VCA survival to >30 days, was unable to induce long-term (>100 days) VCA survival (Fig. 1A), and use of either agent alone led to only 2–3 days of prolongation of allograft survival (data not shown). Therapy with CD154/DST is thought to act primarily by immunomodulatory effects on CD4 T cells; i.v. injection of donor cells is thought to lead to a rapid and synchronous response by recipient lymphoid cells, with upregulation of CD40L/CD154 in 12–24 hours, and the administration of the CD154 mAb is thereby able to efficiently block signal 2 and anergize alloreactive host T cells^6,7^. Given its only modest efficacy in VCA recipients, we considered whether CD8 T cells might promote “breakthrough” rejection of limb VCA in this context. However, mAb depletion of CD8 T cells did not significantly alter the tempo of rejection in recipients treated with CD154/DST or with RPM alone (Fig. 1A). By contrast, adding 4 weeks of RPM therapy (2 mg/kg/d) from the day of engraftment to CD154/DST led to long-term orthotopic VCA survival (>100 days) (Fig. 1B).

### Donor contributions to the optimal efficacy of peri-transplant CD154/DST/RPM therapy

We next considered whether donor bone-marrow cells might be a barrier to limb engraftment by contributing to graft immunogenicity or by promoting graft-versus-host disease. Hence, we irradiated donor mice and on the same day undertook orthotopic hindlimb allografts in conjunction with the CD154 mAb-based COB protocol. To our surprise, donor irradiation abrogated the efficacy of the CD154/DST/RPM protocol and restored acute rejection from 3 weeks post-transplant (Fig. 1C). This was not a toxic effect of irradiation (800 cGy) since it did not affect the long-term survival of C57BL/6 isografts (Fig. 1C). We therefore began to explore the radiation-sensitive components of the donor graft that might be required for the efficacy of our CD154/DST COB protocol. First, we tested the effect of removal of donor bone prior to engraftment. Bone removal resulted in limb allograft rejection despite CD154/DST/RPM therapy, and also accelerated rejection of limbs in mice receiving CD154/DST (Fig. 1D). Together these data suggest that a radiation-sensitive component within the bone-marrow was required for the efficacy of our CD154/DST/RPM protocol.

We next tested the use of donor limbs obtained from Rag1−/− mice that lack mature T or B cells^10^. Again, therapy with CD154/DST/RPM was unable to achieve long-term survival of orthotopic hindlimb allografts when Rag1−/− donors were used (Fig. 1E). Efforts to restore long-term survival in recipients of Rag1−/− donor limbs by i.v. adoptive transfer of 30 × 10^6^ donor splenocytes or donor bone-marrow cells at the time of engraftment were unsuccessful, despite use of CD154/DST/RPM (Fig. 1F). This number of adoptively transferred cells approximates the numbers of bone-marrow cells that we obtained by flushing cells from donor limbs pre-transplant, but the inability to restore long-term VCA acceptance may reflect various factors, including the incorrect or insufficient trafficking of cells and the time it takes to create an immunosuppressive environment in the bone-marrow, especially whilst subject to a developing alloresponse.

Lastly, we tested the effects of donor pre-treatment with AMD3100 (Plerixafor), a CXCR4 inhibitor (CXCR4i) that is used clinically to mobilize bone-marrow cells for subsequent hemopoietic stem cell transplantation^11^. Consistent with the literature, our flow cytometry studies (detailed below) indicated that CXCR4i altered the proportions of multiple cell types including T and B cells from donor bone-marrow^12,13^. Allografts derived from CXCR4i-treated mice (100 µg/d, i.p., on days −4, −2 and on the day of transplantation) underwent acute rejection despite therapy with CD154/DST/RPM (Fig. 1G). Collectively, these data indicate that a CXCR4+ bone-marrow cell component of T or B cell origin is essential for the efficacy of our CD154-based COB protocol.

### Early post-transplant events within the donor BM of recipients treated with CD154/DST/RPM

**Figure 2:**
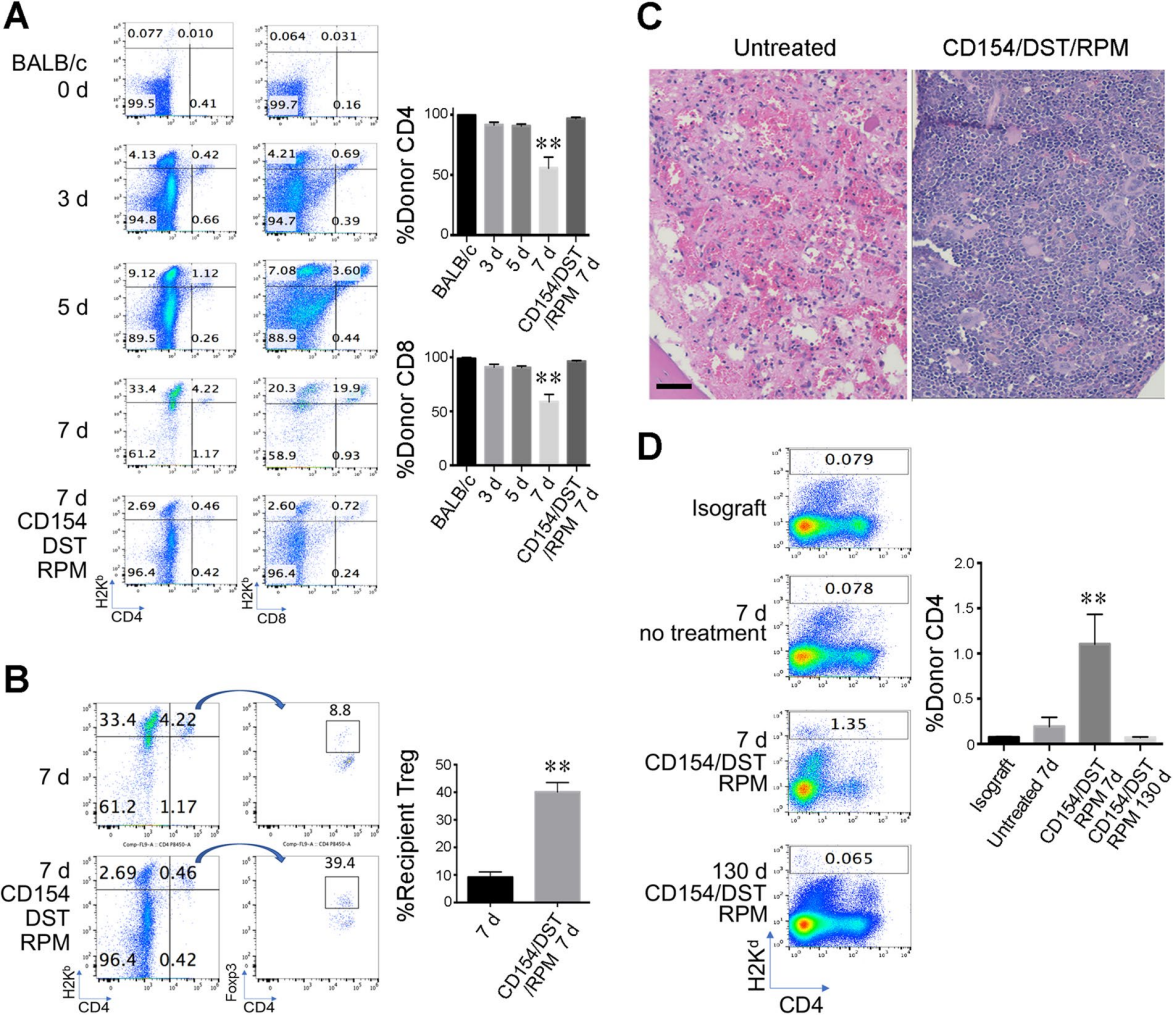
Peri-transplant CD154/RPM therapy results in preservation of donor bone-marrow cells. (**A**) Destruction of donor CD4 and CD8 T cells (BALB/c, H-2^d^) by 7 d post-transplant is prevented by CD154/DST/RPM therapy, with cumulative data from 8 animals/group (mean ± SD) at right. (**B**) Preservation of donor bone-marrow cells by CD154/DST/RPM was associated with an influx of recipient H-2K^b^-positive CD4 + Foxp3+ Treg cells (lower panels); proportions of cells are shown in each panel and are representative of data from 8 recipients, with cumulative data (mean ± SD) at right. (**C**) Histologic images at day 7 posttransplant showing tri-lineage cell preservation *in situ* in CD154/DST/RPM treated allografts, in contrast to the destruction of bone-marrow cells seen in allografts of untreated recipients (bar = 100 µ, representative of 6 allografts/group). (**D**) Peripheral blood samples from VCA recipients treated with CD154/DST/RPM showed increased CD4 T cells at day 7 post-transplant compared to untreated recipients or isograft controls (**p < 0.01) but their proportions were always low (<1–2%) and were not detected in long-surviving allograft recipients (mean ± SD, 6 allografts/group).

Analysis of cells within the donor bone-marrow at 7 days following hindlimb allografting showed decreased small numbers of donor (H-2K^b^-negative) CD4 and CD8 cells in untreated controls, in contrast to their essentially normal numbers in large numbers in recipients receiving CD154/DST/RPM (Fig. 2A). Preservation of donor BM cells was associated with an influx of recipient H-2K^b^-positive CD4+ Foxp3+ Treg cells (Fig. 2B). Differences between the 2 groups were also apparent histologically. The marrow of untreated recipients showed widespread destruction of BM cells, whereas corresponding grafts from mice receiving CD154/DST/RPM showed preservation of bone-marrow cells, including leukocytes, erythroid cells and megakaryocytes (Fig. 2C). Hence, at a time-point at which control grafts have undergone extensive injury and loss of BM cells, the CD154-based COB protocol was found to have preserved tri-lineage bone-marrow cells and was associated with an influx of recipient Treg cells. Despite donor bone-marrow persistence only low proportions of donor cells were demonstrated within the blood of recipient mice, peaking at ~1% at day 7 post-Tx (Fig. 2D), and arguing against micro-chimerism as a dominant mechanism responsible for allograft acceptance in these studies.

### Peritransplant CTLA4Ig/RPM induces long-term VCA survival

**Figure 3:**
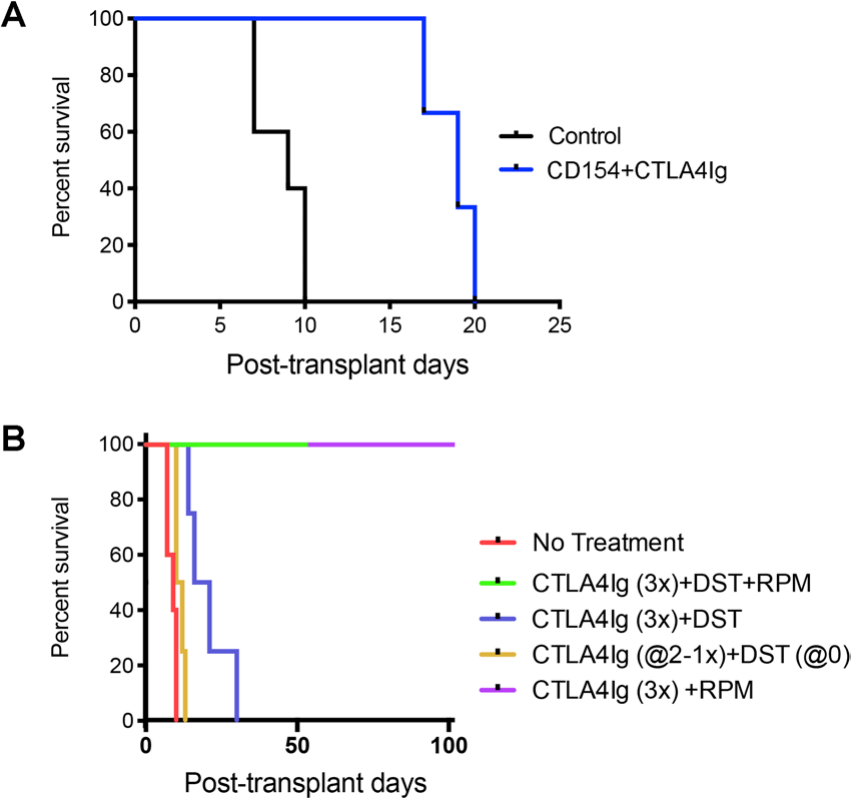
Limited efficacy of CD154 mAb/CTLA4Ig or CTLA4/DST (4 allografts/group) versus CTLA4Ig/RPM; all studies were repeated at least once with comparable results. (**A**) Combined use of peritransplant CD154 mAb (250 µg on days 0, 2 and 4) plus CTLA4Ig (200 µg on days 0, 2, 4 and 6) induced only a doubling of orthotopic VCA survival (*p < 0.05). (**B**) DST (5 × 10^6^ donor splenocytes) at the time of transplantation plus 1 dose of CTLA4Ig (200 µg, i.p.) at day 2 post-transplant had no significant effect on VCA survival. However, 3 doses of CTLA4Ig (200 µg on days 0, 2, 4 and 6) plus DST (5 × 10^6^ on day 0) extended 50% survival to about 3 weeks (*p < 0.05). Addition of RPM (2 mg/kg/d, 4 weeks, Alzet pumps), from the time of engraftment, to CTLA4Ig, with or without added DST, markedly further improved survival, with allografts surviving >100 days (**p < 0.01 vs. CTLA4Ig/DST).

Various forms of CD154 and/or CD40 mAb are in clinical development, but CD154 mAb is not clinically approved. In contrast, a second agent, CTLA4Ig, blocks CD28/B7 interactions and, in the form of Belatacept, is approved for use in human renal transplant recipients. Hence, we examined the effects of peritransplant CTLA4Ig administration, alone or in combination with other agents, on VCA survival. A combination of CD154 (250 µg on days 0, 2 and 4) and CTLA4Ig (200 µg on days 0, 2, 4 and 6), that led to long-term survival of skin and cardiac allografts in the BALB/c-> C57BL/6 combination^14^, led to only a doubling of VCA survival (Fig. 3A). Likewise, a protocol of DST at the time of transplantation plus 1 dose of CTLA4Ig (200 µg, i.p.) at day 2 post-transplant, previously successful in cardiac and renal allograft studies in rodents^15,16^, had no significant effect on VCA survival. However, 3 doses of CTLA4Ig (200 µg on days 0, 2, 4 and 6) plus DST (5 × 10^6^ on day 0) extended 50% survival to about 3 weeks (p < 0.05) (Fig. 3B). Addition of RPM (2 mg/kg/d, 4 weeks, Alzet pumps) to this CTLA4Ig/DST protocol markedly improved survival, with heterotopic allografts surviving >100 days (Fig. 3B). With an eye to clinical translation, this led us to test the effects CTLA4Ig plus RPM, without DST. We found that recipients treated with 3 doses of CTLA4Ig (200 µg on days 0, 2 and 4) plus 28 days of RPM from the time of engraftment maintained their orthotopic allografts for >100 days (Fig. 3B). Hence, CTLA4Ig/RPM is a second peritransplant COB-based protocol that achieves successful engraftment in a stringent VCA model.

### Donor bone-marrow Tregs are essential for the efficacy of peri-transplant CTLA4Ig/RPM therapy

**Figure 4:**
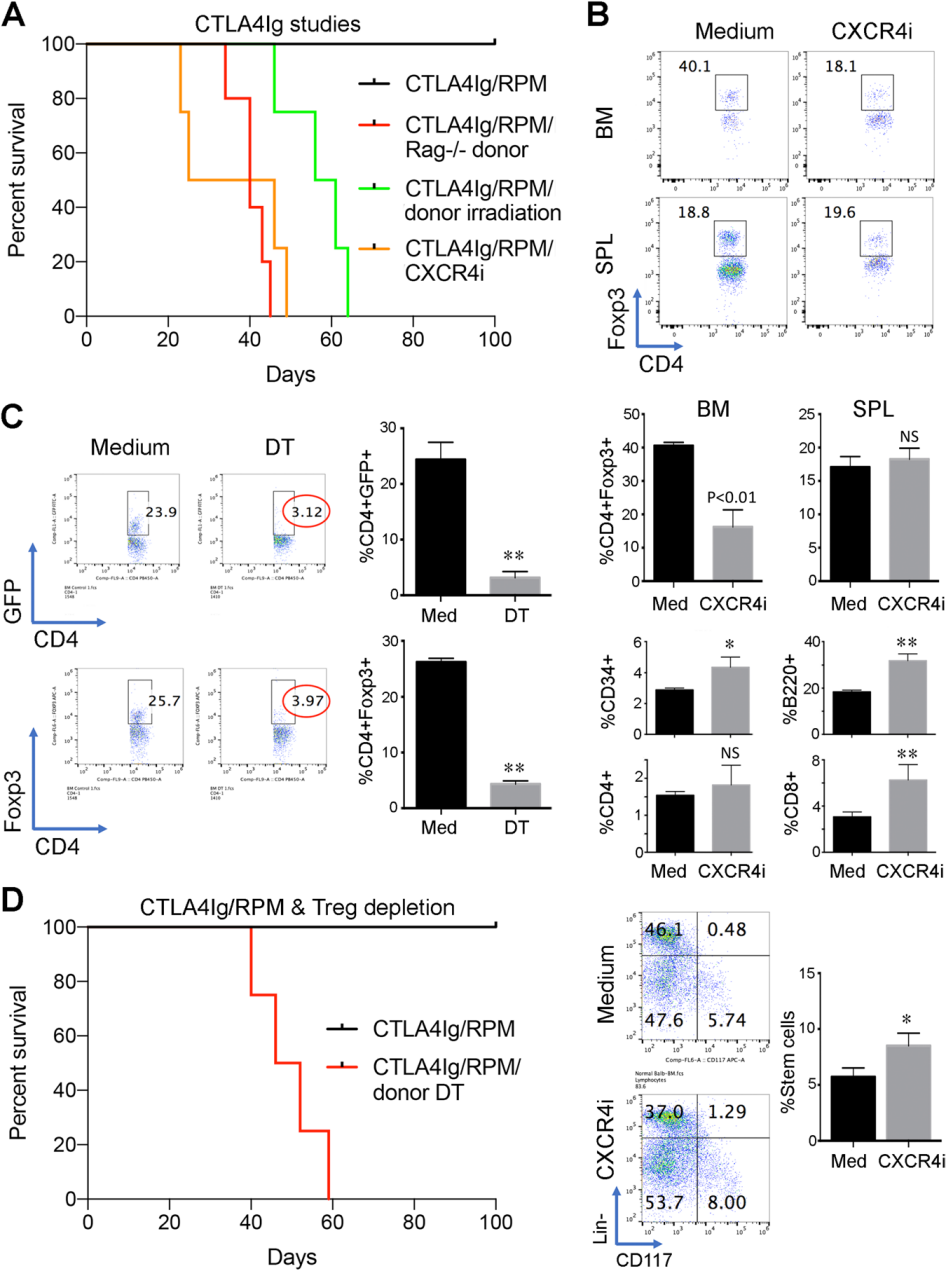
Peri-transplant CTLA4Ig/RPM therapy induces BM Treg-dependent long-term orthotopic hindlimb VCA survival. each Kaplan-Meier plot involves 4 allografts/group and all studies were repeated at least once with comparable results. (**A**) The long-term engraftment (>100 d) achieved using CTLA4Ig/RPM therapy was prevented by use of limbs from irradiated mice (**p < 0.01 vs. non-irradiated donors) or Rag1−/− mice (**p < 0.01 vs. WT donors), or by donor treatment with CXCR4i (**p < 0.01) on days −4, −2 and on the day of transplantation (100 µg/d, i.p.). (**B**) Flow cytometric analysis at the time of transplantation showed that pre-transplant treatment of donor mice with CXCR4i, using the schedule noted in panel A, decreased the donor BM population of Foxp3+ Tregs by >50% (**p < 0.01) but had no effect on splenic (SPL) Treg proportions. CXCR4i therapy increased the proportions of donor BM CD34 + cells (*p < 0.05), B220 + B cells (**p < 0.01), CD8 T cells (**p < 0.01) and lineage-negative (Lin-) CD117 + stem cells (*p < 0.05); representative flow plots are shown along with cumulative data (mean ± SD) from 4 animals/group. (**C**) Pre-transplant treatment of donors with diphtheria toxin (DT) decreased donor BM Treg proportions by 80–90% (**p < 0.01), as shown using DEREG mice whose DT receptor-bearing Tregs also expressed Foxp3 and a GFP transgene; flow plots are representative of 3 separate experiments, with cumulative data (mean ± SD) at right. (**D**) Pre-transplant treatment of donors with DT impaired subsequent VCA survival despite recipient therapy with CTLA4Ig/RPM (**p < 0.01).

We sought to compare the contributions of donor cells in the peritransplant CTLA4Ig/RPM protocol with that seen in our studies with CD154/DST/RPM, described above. As summarized in Fig. 4A, the efficacy of our optimal peri-transplant protocol of CTLA4Ig (200 µg on days 0, 2, 4 and 6) and RPM (2 mg/kg/d, 4 weeks, Alzet pumps) was, as with CD154/DST/RPM therapy, undermined by the use of hindlimbs from (i) donor mice that had undergone pre-transplant irradiation (800 cGy), (ii) use of Rag1−/− donors or (iii) mice receiving pre-transplant therapy with CXCR4i (100 µg/d, i.p., on days −4, −2 and on the day of transplantation).

Our finding that the same factors blocked the efficacy of 2 protocols that are highly effective in prolonging orthotopic VCA survival, CD154/DST/RPM and CTLA4Ig/RPM, led us to focus on which T or B cell population might be required. In murine bone-marrow, as with human bone-marrow, there is a higher frequency of CD4+ CD25+ FoxP3+ Treg cells than in any other secondary lymphoid organs^17^. In addition, bone-marrow reticular and endothelial cells strongly express functional stromal-derived factor (CXCL12), the ligand for CXCR4^18^, and murine and human Tregs traffic to, and are retained in, BM through CXCR4/CXCL12 signals^17^. There is also evidence from hemopoietic stem cell transplant models that the BM may be an immunologically privileged site, whereby donor Tregs can protect allogeneic cells from immune destruction^19^. Hence, we investigated whether Foxp3+ Treg cells present pre-transplant in donor limb bone-marrow are central to the efficacy of COB-based protocols.

Using flow cytometry, we found that use of the CXCR4i in donors pre-transplant led to a > 50% reduction in donor Foxp3+ Tregs within the bone-marrow by the day of transplantation whereas the splenic population of Tregs was unchanged (Fig. 4B). In contrast to the effects of a single injection (not shown), this CXCR4i protocol of 3 injections over several days pre-transplant led to compensatory increases in CD34 + cells, B cells and CD8 T cells within the bone-marrow, as well as inducing expansion of the hemopoietic stem cell (Lin- CD117+) population (Fig. 2B).

To more precisely focus on the donor bone-marrow Treg population, we employed DEREG mice that are engineered to express the diphtheria toxin receptor plus a green fluorescent protein (DTR-eGFP) within fully functional Foxp3 + CD4+ Treg cells^20^. Administration of diphtheria toxin markedly reduced Foxp3+ Tregs in donor bone-marrow pre-transplant (Fig. 4C), and use of these treated donors as hindlimb donors blocked the efficacy of CTLA4Ig/RPM in promoting long-term survival of orthotopic VCA (Fig. 4D). These data show that donor bone-marrow Foxp3+ Tregs are required for the efficacy of this COB-based protocol.

### CXCR4 and Treg functions

**Figure 5:**
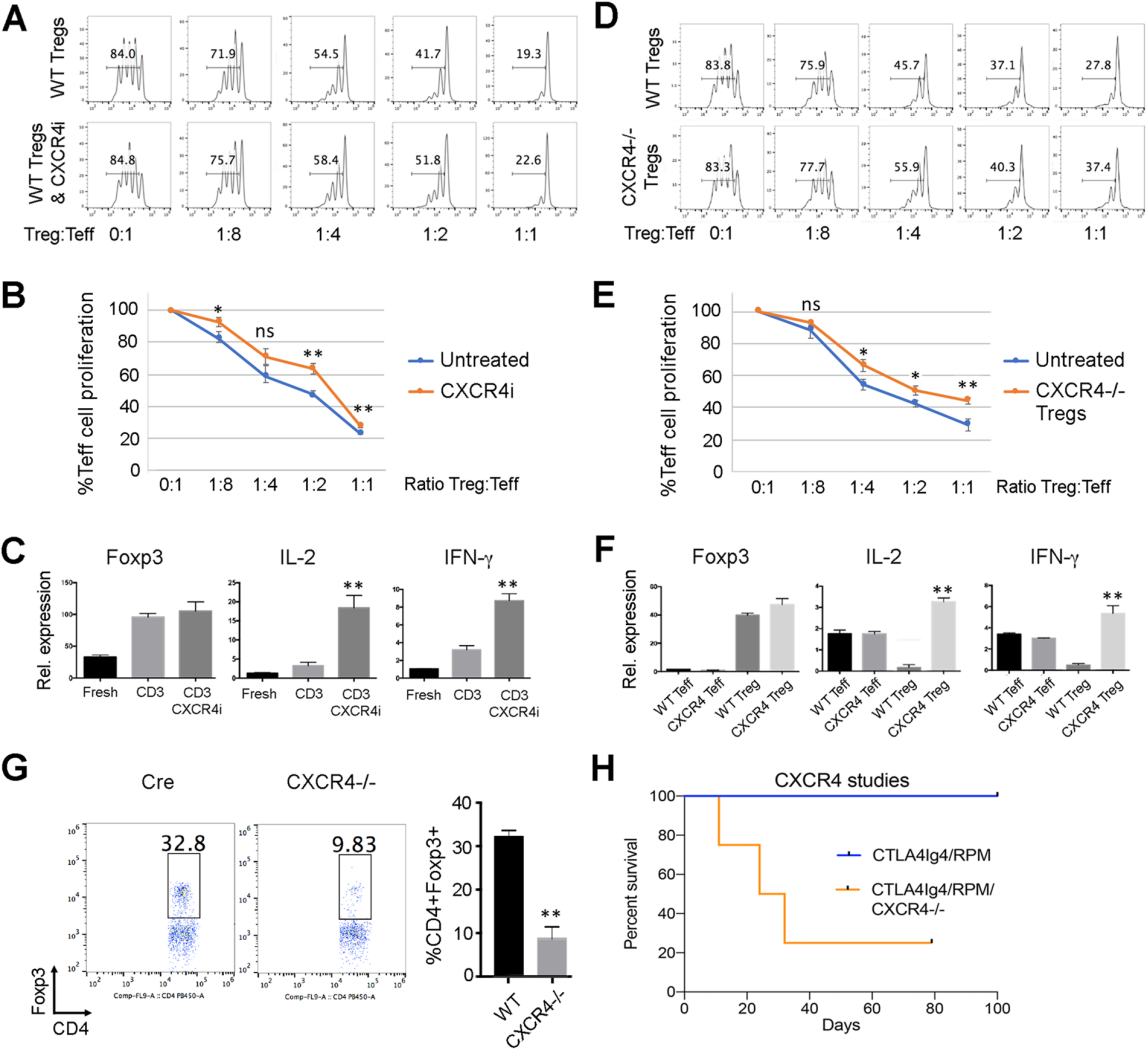
CXCR4 targeting impairs Treg function, alters Treg gene expression and induces VCA rejection. (**A**) Modest impairment of Treg suppressive function *in vitro* using Tregs treated with CXCR4i. Representative of 4 experiments; proportion of proliferating CFSE + CD4 T cells is shown in each panel. (**B**) Cumulative data (mean ± SD) from assays performed in triplicate showing that CXCR4i impairs Treg function *in vitro* (**p < 0.01). (**C**) CXCR4i increases IL-2 and IFN-γ mRNA expression by Tregs that were freshly isolated and then cultured overnight in the presence of CD3 mAb with or without added CXCR4i (3 wells/group, **p < 0.01 vs. CD3 alone; representative of 2 experiments). (**D**) Modestly impaired Treg suppressive function of CXCR4−/− vs. WT Tregs, representative of 4 experiments. (**E**) Cumulative data (mean ± SD) from assays performed in triplicate showing that CXCR4 deletion impairs Treg function *in vitro* (*p < 0.05, **p < 0.01). (F) Conditional CXCR4 deletion in Tregs led to increased IL-2 and IFN-γ production by freshly isolated Tregs (n = 3 mice/group at 6 weeks of age, **p < 0.01 vs. WT Treg cells). (**G**) Conditional deletion of CXCR4 in Foxp3+ Treg cells decreased the proportion of BM Tregs by ~70%, as shown by flow cytometric evaluation of BM cells from mice at 6 weeks of age, with cumulative data at right (mean ± SD, 4/group, **p < 0.01). (H) Conditional deletion of CXCR4 in donor Foxp3+ Treg cells resulted in the failure of peri-transplant CTLA4Ig/RPM therapy to induce long-term VCA survival (4 allografts/group, **p < 0.01); this experiment was repeated once with comparable results.

Lastly, we considered whether Treg expression of CXCR4 might serve one or more roles beyond simply being responsible for the bone-marrow accumulation of Treg cells^17^. Treatment of Tregs with the CXCR4i, AMD3100, led to mildly impaired Treg suppressive function *in vitro* (Fig. 5A,B), i.e. in a context in which chemotaxis is likely irrelevant since the Tregs and Teff cells are side-by-side. Analysis of gene expression by Tregs under resting vs. activating conditions *in vitro* showed that CXCR4i promoted Treg expression of IL-2 and IFN-γ (Fig. 5C), consistent with Treg dysregulation since Foxp3 normally prevents the expression of these effector cytokine genes in Treg cells^21^. We then conditionally deleted CXCR4 in Tregs by mating Foxp3^YFP-Cre^ mice and CXCR4^fl/fl^ mice. Compared to WT Treg cells, purified CXCR4−/− Tregs showed reduced Treg suppressive function (Fig. 5D,E), and increased expression of IL-2 and IFN-γ mRNAs (Fig. 5F). These mice had markedly reduced proportions of Foxp3+ Tregs in their hindlimb bone-marrow compartments (Fig. 5G), and their use as limb donors led to impaired VCA survival despite therapy with CTLA4Ig/RPM (Fig. 5H). These findings indicate that CXCR4 is required for normal accumulation of Tregs in donor bone-marrow, but likely also has one or more additional functions beyond chemoattraction since conditional deletion or use of a CXCR4i leads to decreased Treg suppressive function and abnormal Treg cytokine production of IL-2 and IFN-γ.

**Figure 6:**
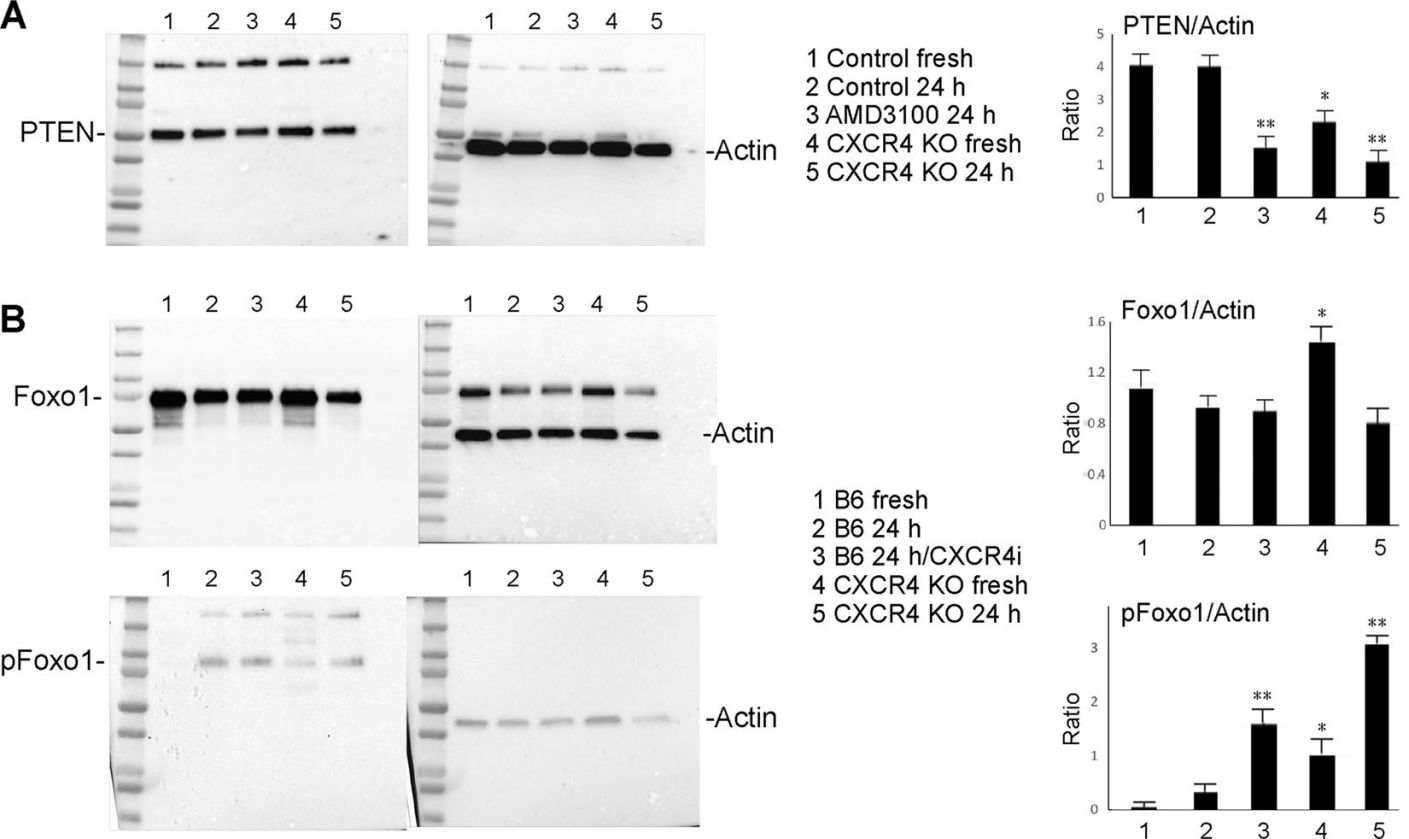
Without tonic CXCR4 signaling, Tregs downregulate PTEN and increase pFoxo1 generation. (**A**) Western blot studies showed PTEN levels were maintained upon activation of WT Tregs but were impaired by treatment with CXCR4i, and similarly, CXCR4−/− Tregs had less PTEN expression than WT Tregs and levels were further reduced upon Treg activation. Quantitative data from 3 samples/group are shown at right as the ratio of PTEN to beta-actin. (**B**) Consistent with decreased PTEN expression by CXCR4−/− Tregs, phospho-Foxo1 levels were increased by CXCR4i treatment of Tregs undergoing activation, and levels were increased in CXCR4−/− vs. WT Tregs and rose still higher upon cell activation. Quantitative data from 3 samples/group are shown at right as the ratio of Foxo1 to beta-actin, and phosphor-Foxo1 to beta-actin. Statistical analysis by 1-way ANOVA, *p < 0.05 and **p < 0.01 involving in panel A (PTEN), group 3 vs. group 2, group 4 vs. group 1, and group 5 vs. group 2; and in panel B (Foxo1) group 4 vs. group 1, and for p-Foxo1, group 3 vs. group 2, group 4 vs. group 1, and group 5 vs. group 2. Original uncut gels are shown in the Supplement.

Treg homeostasis and stability are regulated by their expression of phosphatase and tensin homolog (PTEN), which suppresses phosphoinositide 3-kinase (PI3K) and Akt signaling^22,23^, including Foxo1 phosphorylation by Akt^24,25^. Western blot studies showed PTEN levels were maintained upon activation of WT Tregs but were impaired by treatment with CXCR4i (Fig. 6A), and similarly, CXCR4−/− Tregs had less PTEN expression than WT Tregs and levels were further reduced upon Treg activation (Fig. 6A). Consistent with decreased PTEN expression by CXCR4 targeted Tregs, phospho-Foxo1 levels were increased by CXCR4i treatment of Tregs undergoing activation, and levels were increased in CXCR4−/− vs. WT Tregs and rose still higher upon cell activation (Fig. 6B). These data suggest that without tonic CXCR4 signaling, Tregs downregulate PTEN and increase Akt-mediated pFoxo1 generation.

## Discussion

Given that well over a decade of clinical trials with Tregs as adoptive cell therapy in transplant recipients has not produced any compelling successes, it may be time to reconsider how best to exploit Treg biology in the context of transplantation. In particular, physiologic levels of Foxp3+ Tregs may be sufficient to maintain allograft survival following various therapeutic interventions, involving “Treg-friendly” COB and/or RPM, and the avoidance of calcineurin inhibitor-based protocols so as to allow optimal Treg functioning^5^. While the many reports of the efficacy of costimulation blockade in experimental transplant models have not led to corresponding success clinically, COB using a modified form of CTLA4Ig (Belatacept) has utility in curtailing alloresponses and avoiding calcineurin inhibitor use in renal transplant recipients, thereby helping preserve glomerular filtration rates long-term^26,27^. Whether ongoing Belatacept therapy impairs Treg function long-term by blocking the inhibitory actions of CTLA4 remains unclear^28,29^, though its use clinically does require monthly infusion. A critical role for chemokine-dependent recruitment of Tregs to allografts in mice undergoing COB has been reported^8^, but there are no data on donor Tregs being required for successful engraftment of solid organs or VCA tissues. Hence, the current studies are significant in showing how the efficacy of 2 different peri-transplant COB protocols both depended upon the presence of Foxp3+ Treg cells present within donor bone-marrow.

Osteoblasts, endothelial cells and reticular cells within the BM are the major sources of CXCL12 production^30^, the cognate ligand for the G protein-coupled receptor, CXCR4, which is expressed by mononuclear leukocytes and bone-marrow progenitor cells. This ongoing production of CXL12 in the BM leads to Treg recruitment and immobilization; there are greater than 2-fold more Tregs in the human BM than in the thymus, blood or lymph nodes, and these bone-marrow Treg cells express more Foxp3 and have greater suppressive activity than non-bone-marrow Treg cells^17^. Foxp3+ Treg cells are thought to maintain hemopoietic stem cell (HSC) quiescence by their generation of adenosine, and this quiescent state hence promotes HSC longevity^31^. Components of this large Treg reservoir within the bone-marrow are thereby present during limb VCA procedures. We found that donor bone-marrow cells are destroyed by alloreactive host cells in the first week post-transplant but are preserved in mice receiving COB, and we observed accumulation of recipient Tregs within the donor bone-marrow. The spatial and contextual details of the interactions of donor and recipient cells within the bone-marrow compartments of limb allografts remain to be dissected, just as the details of the concept that this site is immunologically privileged remains to be elucidated^19^. However, it might provide a site at which alloreactive host Teff cells undergo immunomodulation by resident Foxp3+ Treg cells.

Our studies also suggest that beyond promoting the bone-marrow accumulation of Tregs, CXCL12 signaling through the CXCR4 receptor may have important effects in terms of Treg homeostasis and stability, since targeting of the receptor impaired Treg function, decreased PTEN expression and increased phosphorylation of Foxo1. These findings are consistent with studies of signaling within cancer cells^32^, and with the utility of CXCR4 targeting in disrupting the functions of tumor associated Treg cells^33–35^. Foxo1 is a transcription factor essential for control of the expression of Foxp3 and its target genes; phosphorylation of Foxo1 leads to its nuclear export and Treg dysfunction. Hence, the local production of CXCL12 by cells within bone-marrow niches promotes Treg accumulation and also stabilizes their function^24,25^.

On a practical level, further studies are required to image serial events within the donor bone-marrow post-transplant, as well as to test aspects of host acceptance of the grafts. We still need insights into whether host alloreactive T cells are being suppressed by donor Tregs within the donor bone-marrow or as a result of donor Treg migration beyond their bone-marrow niche. Of particular interest will be the assessment of whether donor-specific tolerance can be achieved, the relative contribution of donor vs. recipient bone-marrow cells (including Tregs) in long-surviving donor long-bones as well as in recipient bone-marrow, and the potential for ongoing freedom from the development of chronic rejection at periods considerably later than 100 days post-transplant. However, the current studies already suggest optimism in seeking to develop therapeutic protocols for VCA procedures that involve donor BM, given the ability to administer clinically approved agents, such a CTLA4Ig and RPM, in the peri-transplant period and achieve sustained engraftment without maintenance immunosuppression. Hence, there may be better and less toxic ways to immunosuppress patients after VCA than the current calcineurin inhibitor-based protocols, and the data from experimental models may be especially relevant given that the small numbers of patients currently undergoing VCA procedures do not lend themselves to extensive clinical experimentation.

## Materials and Methods

### Mice

We purchased WT BALB/c and WT C57BL/6 mice; B6/Rag−/− mice lacking mature T and B cells^10^; DEREG/B6 mice engineered to express the diphtheria toxin receptor plus a green fluorescent protein (DTR-eGFP) within fully functional Foxp3 + CD4 + Treg cells^20^; Foxp3^YFP-Cre^ mice^36^ and CXCR4^fl/fl^ mice^37^ (both strains on the B6 background) from The Jackson Laboratory. All mice were used at 8–12 weeks of age. Animal study protocols were approved and undertaken in accordance with the regulations and guidelines of the Institutional Animal Care and Use Committee of The Children’s Hospital of Philadelphia (19-001052). Experiments were performed with age‐ and sex‐matched mice and using animals that were littermates or were maintained in the same room and/or were co‐housed within the same cages for >2 weeks to limit potential effects of microbiome differences.

### Heterotopic and orthotopic hindlimb transplantation

Heterotopic and orthotopic hindlimb transplants were performed using BALB/c donors and C57BL/6 recipients, as described^38^, except that the anastomoses of donor and recipient blood vessels were performed by direct suturing rather than utilizing a cuff technique. Nerve sheaths were anastomosed by direct suturing. Mice were examined daily for signs of vascular compromise and rejection; the latter was apparent as hair loss, swelling and erythema of the skin that rapidly progressed to skin necrosis and was confirmed by histology^38^.

### Reagents

We purchased CXCR4i (AMD3100, Sigma-Aldrich); CD8 mAb (clone YTS 169.4), CD154 mAb (clone MR1) and murine CTLA4-Ig (BioXcell); diphtheria toxin (Sigma-Aldrich) and RPM (Sigma-Aldrich).

### Donor treatments

In some experiments, WT BALB/c mice were subjected to pre-Tx whole-body irradiation (800 cGy), on the same day as their use as limb transplant donors. DEREG mice were injected daily for 6 days with diphtheria toxin (DT, 1 µg/injection, i.p.) to deplete Tregs prior to their use as limb transplant donors. Additional donor mice were treated with AMD3100, a CXCR4 inhibitor (CXCR4i), on days −4, −2 and on the day of transplantation (100 µg/d, i.p.).

### Recipient treatments

Recipients were treated with CD154 mAb (clone MR1, 200 μg, BioXcell) with or without donor splenocyte transfusion (DST, 5 × 10^6^ cells), i.v., at transplantation, and in some series, mice also received RPM (2 mg/kg/d) delivered by a 4-week Alzet osmotic pumps inserted at transplantation. In additional studies, recipients were treated with murine CTLA4‐Ig (BioXcell, 500 μg), i.p., at days 0, 2 and 4 post-Tx, alone or in conjunction with RPM (2 mg/kg/d) delivered by a 4-week Alzet pumps inserted at transplantation. Recipient CD8 T cells were depleted by weekly i.p. injection of CD8a mAb (BioXcell, clone YTS-169.4, 500 µg) until rejection^39^.

### Treg isolation and suppression assays

*In vitro* studies were performed using cell-sorted CD4 + YFP + Tregs or CD4 + CD25+ Tregs isolated with magnetic beads from Foxp3^YFP-Cre^ mice and equal numbers of antigen-presenting cells and Teff cells; experimental protocols and data analysis were recently described in detail^40^.

### Antibodies and flow cytometry

We purchased mAbs directed against murine CD4 (BD Bioscience, Pacific blue, clone RM4-5, #558107), CD8 (PE-Cy7, eBioscience, clone 53-6.7, #25-0081), Foxp3 (PE-Cy5, eBioscience, clone FJK-16s, #15-5773), CD25 (APC, eBioscience, clone PC61.5, #17-0251), H-2K^b^ (APC, BD Bioscience, clone AF6-88.5), H-2K^d^ (APC, BD Bioscience, clone SF1-1.1), CD34 (FITC, BD Bioscience, clone RAM34) and Lineage Antibody Cocktail (APC, BD Bioscience), and performed flow cytometry using a CyAn flow cytometer (Beckman Coulter, Brea, CA). We also purchased unconjugated CD3 (clone 145-2C11, #553057) and CD28 (clone 37.51, #553294) mAbs from BD Bioscience.

### Histology

Limb specimens were fixed in 10% neutral buffered formalin, decalcified in Formical-2000, and embedded in paraffin. Histologic sections (6 μM) were stained with hematoxylin and eosin, reviewed by a pathologist (W.W.H) blinded to conditions, and graded using Banff criteria^41^.

### Real-time qPCR

RNA was isolated using RNeasy kits (Qiagen), with RNA integrity and quantity analysis using a NanoDrop ND-1000 and a Nanochip 2100 Bioanalyzer (Agilent Technologies)^9^; qPCR was performed using Taqman primer and probe sets, with data normalized to 18 s rRNA and relative expression determined by the formula 2^−ΔCT^.

### Western blotting

Western blots were performed using Abs directed against Foxo1, phospho-Foxo1, PTEN (eBioscience) and β-actin (Cell Signaling Technology)^9^.

### Statistics

We used GraphPad Prism to analyze data. Differences between two groups were assessed with a 2-tailed Student’s t-test if data were normally distributed, or Mann-Whitney U unpaired test if not normally distributed. Groups of three or more were analyzed by 1-way ANOVA, with Tukey’s multiple comparison test if normally distributed, or the Kruskal-Wallis with Dunn’s multiple comparison test if not normally distributed. Graft survival was evaluated with Kaplan-Meier followed by log-rank test. P < 0.05 was considered significant.

### Contact for Reagent and Resource Sharing

Further information and requests for resources and reagents should be directed and will be fulfilled by the Lead Contact, Wayne W. Hancock (whancock@pennmedicine.upenn.edu).

## Supplementary information

Supplementary information.
