# Supplementary material for: Donor bone-marrow CXCR4+ Foxp3+ T-regulatory cells are essential for costimulation blockade-induced long-term survival of murine limb transplants
Source: Sci Rep. 2020 Jun 9;10:9292. doi: 10.1038/s41598-020-66139-x (PMC7283338; doi:10.1038/s41598-020-66139-x)

Supplement: Original Uncut Gels

Donor bone-marrow CXCR4+ Foxp3+ T-regulatory cells are essential for costimulation blockade-induced long-term survival of murine limb transplants

Liqing Wang, Zhonglin Wang, Rongxiang Han, Arabinda Samanta, Guanghui Ge, L. Scott Levin, Matthew H. Levine, Wayne W. Hancock

Uncut blots for Figure 6

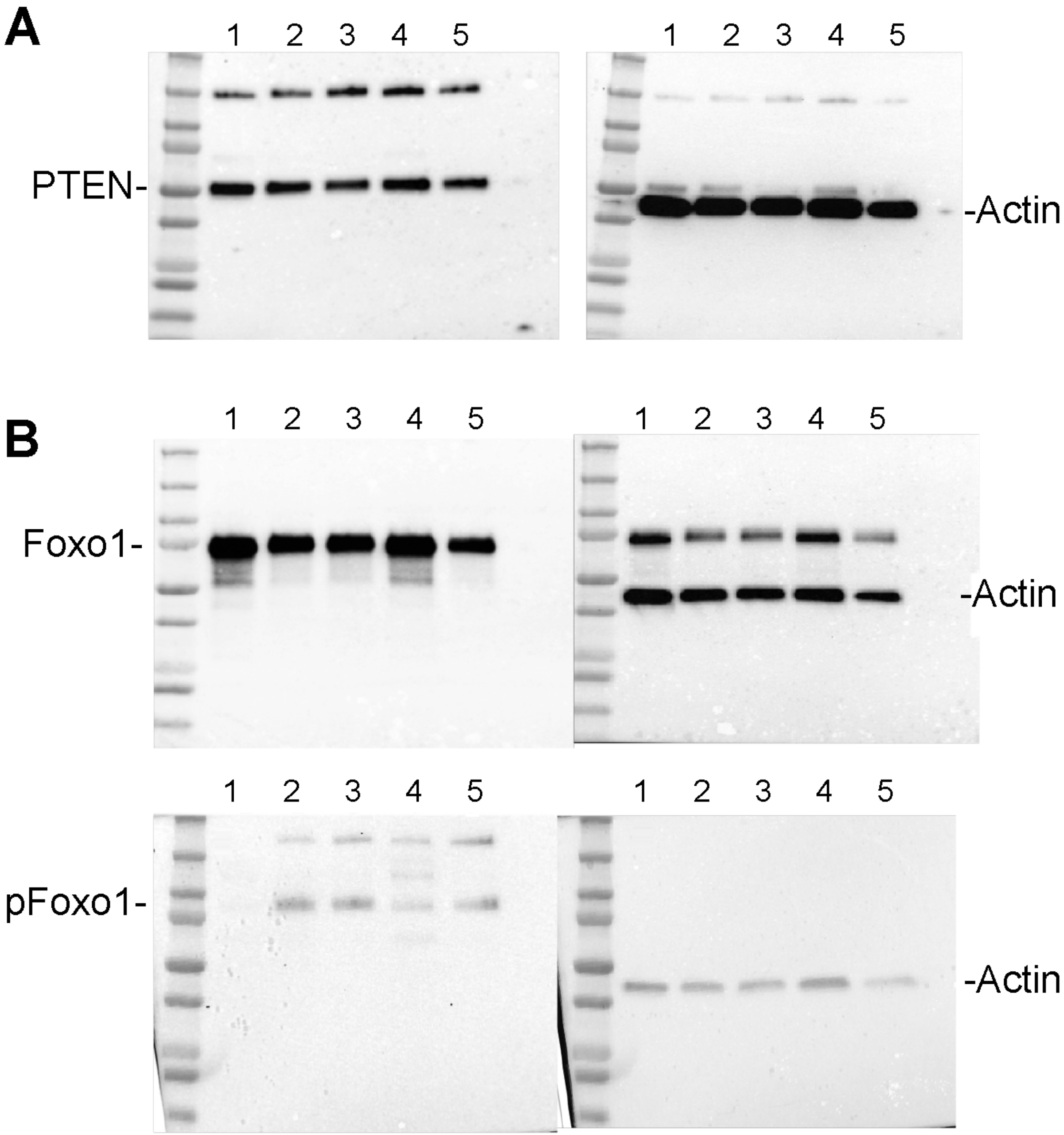

Uncut blots for Figure 6
(less contrast)

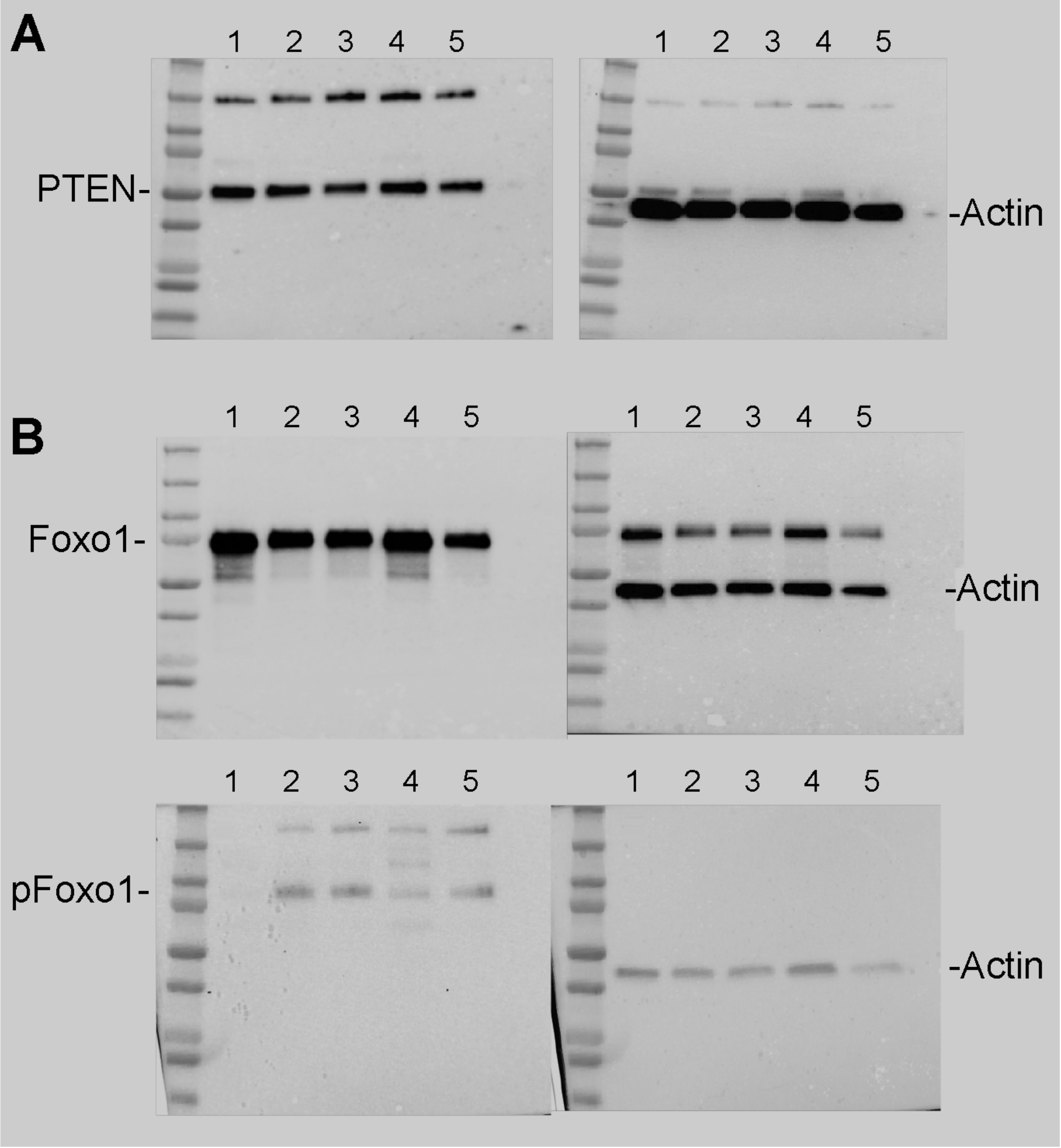

Supplement: Supplementary file 1 — Supplementary information. [file 41598_2020_66139_MOESM1_ESM.pdf]
